# Genome-Wide Identification and Analysis of the *JAZ* Gene Family in *Artemisia argyi*

**DOI:** 10.3390/cimb47020100

**Published:** 2025-02-06

**Authors:** Zhanbin Gong, Xueshuang Wu, Yilin Luo, Tianhong Zhou, Zhenchao Yang, Yongjun Wu

**Affiliations:** 1 College of Life Sciences, Northwest A & F University, Yangling 712000, China; gzb0815@nwafu.edu.cn (Z.G.); wuxueshuang@nwafu.edu.cn (X.W.); luoyilin@nwafu.edu.cn (Y.L.); zhouth@nwafu.edu.cn (T.Z.); 2 Key Laboratory of Northwest Facility Horticulture Engineering of Ministry of Agriculture and Rural Affairs, College of Horticulture, Northwest A & F University, Yangling 712000, China

**Keywords:** genome-wide analysis, *Artemisia argyi*, *JAZ* gene family, expression analysis

## Abstract

*Artemisia argyi* H. Lév. & Vaniot (*A. argyi*) is a perennial herb belonging to the Asteraceae family and is a medicinal plant widely used in traditional medicine. In the field of plant physiology, JAZ proteins play a central role in the jasmonic acid (JA) signaling pathway, significantly affecting plant growth and development as well as responses to biotic and abiotic stresses. This study aims to identify and analyze the *JAZ* gene family of *A. argyi*. Through a genome-wide analysis of *A. argyi*. 18 *JAZ* genes were identified and classified into three subfamilies, based on phylogenetic relationships. Additionally, for this study, we comprehensively analyzed the physical and chemical properties, gene structure, chromosomal locations, conserved domains, cis-acting elements, and evolutionary relationships of these genes. The tissue-specific expression patterns of *JAZ* genes were obtained from transcriptome data, revealing distinct expression profiles across different tissues in *A. argyi*. Finally, this research identified a candidate *JAZ* gene, *AarJAZ18*, which is involved in the development of glandular trichomes in the leaves of *A. argyi*. Subsequently, the relative expression levels of *AarJAZ18* in different tissues were validated using quantitative real-time PCR (qRT-PCR). In summary, this study provides a foundation for further investigation into the functions of *A. argyi JAZ* genes and offers valuable gene resources for breeding superior varieties and enhancing germplasm innovation.

## 1. Introduction

Jasmonic acid is an important plant hormone, and jasmonates (JAs), including jasmonic acid and its bioactive derivatives, play a significant role in the plant’s response to biotic and abiotic stresses, as well as in plant growth and development [1,2,3]. In plants, JA is primarily produced through the oxygenated phospholipid biosynthesis pathway, which originates from the α-linolenic acid released from membrane lipids. The JAZ protein family comes from a large protein family known as TIFY, which was previously named ZIM [4]. The TIFY family also includes three other subfamilies: TIFY, PPD, and ZML [4,5]. All sub-families contain a conserved TIFY domain, as well as one or two different additional domains. The JAZ protein family consists of two highly conserved functional domains, namely, the TIFY (also known as ZIM) and Jas (also known as CCT_2) domains [6]. The TIFY domain (PF06200) typically consists of 28 amino acids and has a conserved sequence “TIF[F/Y]XG” [4], located at the N-terminal of the JAZ protein sequence. There are also some TIFY domain variants, like TMLYGG, SFFYGG, TIFYNG, and TIFING [7]. The Jas domain (PF09425) is characterized by a “SLX2FX2KRX2RX5PY” motif, a variant of the CCT domain [8,9], located at the C-terminal of the JAZ protein sequence. Both the TIFY and Jas domains, which are highly conserved in JAZ proteins, are crucial in the JA signaling pathway. The TIFY domain mediates the interaction between JAZ proteins and the NINJA-TPL complex, thereby cooperatively suppressing the signal transduction of the JA pathway [10]. Additionally, the Jas domain mediates the interaction between *JAZ* and other genes (such as *COI1* and *MYC*), thereby inhibiting their transcriptional activity [11,12,13]. Therefore, JAZ proteins are vital in the JA-triggered signaling cascade.

In 2007, JAZ proteins were first discovered in *Arabidopsis thaliana* [6]. Since then, with the rapid development of gene sequencing technology, an increasing number of plant *JAZ* genes have been identified. For example, 12, 15, 9, 26, and 23 *JAZ* genes have been found in *Populus trichocarpa* [14], *Oryza sativa* [15], *Artemisia annua* [16], *Solanum lycopersicum* [17], and *Zea mays* [18], respectively. As more *JAZ* genes are identified, their functions in plant resistance to biotic and abiotic stresses and in regulating plant development are gradually being uncovered. For instance, Arabidopsis *AtJAZ4* is involved in regulating the development of roots, hypocotyls, and petioles [19]; *OsJAZ9* in *O. sativa* may participate in tolerance to water deficit stress by regulating the width of *O. sativa* leaves and stomatal density [20]; *VqJAZ7* in grapevines can enhance resistance to powdery mildew by regulating cell death and the accumulation of superoxide anions [21]; and the overexpression of *GsJAZ2* in soybean significantly enhances the transgenic lines’ resistance to salt stress [22]. These pieces of evidence all indicate that *JAZ* genes play an important role in plant growth and development, as well as in stress resistance.

*A. argyi* is a perennial herbaceous plant in the Asteraceae family, with over three hundred species that are widely distributed in Asian countries such as China, Korea, and Japan [23]. As a perennial herbaceous plant, its lifespan can reach 3–5 years in suitable environments. Compared to the harsh growing conditions of some other medicinal plants, *A. argyi* can grow in a variety of habitats, including grasslands, hillsides, and along riverbanks, thriving in temperate regions with well-drained soils. For thousands of years, *A. argyi* has been extensively used in traditional Chinese medicine, both internally and externally, for treating conditions such as dysmenorrhea, abdominal pain, and inflammation [24]. These medicinal values are inseparable from the rich bioactive components in *A. argyi* leaves, mainly including polysaccharides, flavonoids, alkaloids, and volatile oils [25]. *A. argyi* glandular trichomes have been found to be the site of the synthesis and storage of volatile oils, as well as the key tissue in the production of flavonoids and terpenoids [26,27]. In recent years, due to factors such as long-term monocropping, pest and disease infestations, genetic diversity issues, and improper management, some cultivation areas have experienced a decline in the yield and quality of *A. argyi*, significantly impacting both its quality and production. Therefore, it is imperative to initiate genetic research aimed at identifying those genes that can contribute to the enhancement of high-quality core *A. argyi* cultivars. However, most of the current research on *A. argyi* is limited to its chemical composition, pharmacological effects, and bioactivity [28,29,30]. Through previous studies [14,15,16,17,18,19,20,21,22], we believe that the *JAZ* gene also plays an important role in the growth and development of *A. argyi*. Therefore, based on the reported *A. argyi* genomic data, this study identified those members of the *JAZ* gene family in *A. argyi* and analyzed their functions through bioinformatics, identifying a total of 18 *A. argyi JAZ* genes and uncovering a *JAZ* gene that may affect the development of *A. argyi* glandular trichomes. This provides a theoretical basis for further research on molecular biology in *A. argyi* and some potential genetic resources for the creation of superior *A. argyi* varieties in the future.

## 2. Materials and Methods

### 2.1. Identification and Physicochemical Properties Analysis of JAZ Genes in A. argyi

In this study, we obtained the whole-genome data and annotation files for *A. argyi* and *A*. *thaliana* from the *A. argyi* Gene Database [31] (https://ngdc.cncb.ac.cn/search/?dbId=gwh&q=PRJCA010808&page=, accessed on 14 September 2024) and the TAIR *Arabidopsis* Genome Database (https://www.arabidopsis.org, accessed on 14 September 2024), respectively. To comprehensively identify the members of the *A. argyi JAZ* gene family, we employed two strategies. First, bidirectional BLAST analysis was performed using the *Arabidopsis* JAZ protein sequences as queries to identify corresponding *A. argyi* JAZ protein sequences with similar structural characteristics, with a minimum hit e-value = 1e–5. Second, we downloaded HMM-format files containing the conserved domains of TIFY and Jas (accession numbers PF06200 and PF09425, respectively) from the Pfam database. The Simple HMM search function in TBtoolsv2.056 was then used to identify *A. argyi* protein sequences containing both conserved domains, where the minimum hit e-value = 0.01.

### 2.2. Physicochemical Properties Analysis of JAZ Genes in A. argyi

The physicochemical properties of each candidate gene, including protein length, isoelectric point, hydrophobicity, and instability index, were analyzed using the ExPASy ProtParam tool (https://web.expasy.org/cgi-bin/protparam/protparam, accessed on 19 September 2024) [32]. Additionally, the subcellular localization of the proteins was predicted using Plant-mPLoc (http://www.csbio.sjtu.edu.cn, accessed on 19 September 2024) by providing the protein sequences.

### 2.3. Phylogenetic Analysis of JAZ Genes in A. argyi

To perform the phylogenetic analysis, JAZ protein sequences from dicotyledonous plants, including the model species *A. thaliana* and *Artemisia annua*, were retrieved from the NCBI database. In addition, JAZ protein sequences from monocotyledonous plants such as *O. sativa* and *Z. mays* were also obtained. Detailed sequence information is provided in Table S4. These sequences, together with the identified JAZ protein sequences from *A. argyi*, were introduced into MEGA11 software for multiple sequence alignment using the MUSCLE algorithm [33]. For phylogenetic analysis, the neighbor-joining method was applied, using the most appropriate substitution model and 1000 bootstrap replicates. The phylogenetic tree was visualized and enhanced using EvolView (http://www.evolgenius.info/evolview, accessed on 19 September 2024) [34].

### 2.4. Gene Structure and Conservative Domain Analysis of JAZ Genes in A. argyi

Gene structure analysis was performed using the *A. argyi* genome annotation data in GFF format. TBtools was employed to analyze the intron-exon structures of the 18 identified *JAZ* genes. For further functional analysis of *AarJAZs*, the protein sequences were submitted to the MEME suite (https://meme-suite.org/meme/doc, accessed on 20 September 2024) to identify conserved motifs. The search parameters included a maximum of 10 motifs, with motif repetitions set to 0 or 1. The motif data from MEME, along with the gene structure and conserved domains, was integrated into a composite diagram using TBtools. This diagram includes the phylogenetic tree, conserved domains, gene structure, and motifs, facilitating a deeper understanding of the structural features and functional implications of the *JAZ* genes in *A. argyi*.

### 2.5. Gene Duplication and Collinearity Analysis of JAZ Family Genes in A. argyi

Gene density analysis of the *A. argyi* genome was conducted using TBtools, and the results were visualized on chromosomes. The positions of the *AarJAZ* genes were determined using the genome annotation file, and a distribution map of all *AarJAZ* genes was generated. Gene duplication and collinearity analysis of the *JAZ* family members were performed using the One-Step MCScanX function in TBtools. The chromosomal distribution and segmental duplication events of the 18 *JAZ* genes in *A. argyi* were visualized using the Advanced Circos function. Additionally, genome data and annotation files for *A. thaliana*, *O. sativa*, *Z. mays*, and *A. annua* were retrieved from the NCBI database (https://www.ncbi.nlm.nih.gov/genome, accessed on 25 September 2024). To examine the evolutionary relationships of segmental duplication events, the Ka, Ks, and Ka/Ks values of the segmental duplication gene pairs were calculated using the Simple Ka/Ks Calculator function in TBtools.

### 2.6. GO Enrichment Analysis of JAZ Genes in A. argyi

The 18 JAZ protein sequences of *A. argyi* were submitted to eggNOG-mapper (http://eggnog-mapper.embl.de, accessed on 29 September 2024) for GO enrichment annotation [35]. The analysis was performed with the following parameters: minimum hit e-value = 0.001, minimum hit bit-score = 60, percentage identity = 40. The annotation data were then processed and visualized using TBtools software.

### 2.7. Expression Analysis of JAZ Genes in A. argyi

The RNA-seq expression profiles of *AarJAZs* were mined from the transcriptome data by Cui et al. [36]. Expression heat maps were generated using TBtools software and row scaling was performed. The *A. argyi* plants used in this study were sourced from Qichun County, Hubei Province, in 2023. The collected roots, stems, and leaves of *A. argyi* were grown in a light-controlled incubator under conditions of 25 °C and a 16/8-h light/dark cycle. During the active growth phase, samples from the roots, stems, and leaves were immediately frozen in liquid nitrogen and stored at −80 °C for later use. Following sample collection, tissues were rapidly frozen in liquid nitrogen, and primers were designed based on sequence specificity. Total RNA was extracted using Trizol (Sangon), and cDNA synthesis was performed using a cDNA reverse transcription kit with 1 μg of RNA. For qRT-PCR analysis, each result was derived from independent RNA samples, with three biological replicates and three technical replicates per experiment, and the relative expression levels of transcripts were calculated via the 2−∆∆Ct method. The *A. argyi* actin gene was used as an internal control for each PCR experiment. The primers used for the qRT-PCR analysis are listed in Table S5.

## 3. Results

### 3.1. Identification and Characterization of JAZ Genes in A. argyi

This study employed two retrieval strategies to identify sequences containing the TIFY and Jas domains within the genome database of *A. argyi*, ultimately identifying 33 candidate *A. argyi JAZ* gene sequences. After removing redundant entries and verifying the presence of conserved domains, 18 distinct *JAZ* gene sequences were confirmed in *A. argyi*, with the full-length protein sequences provided in Table S1. The sequences were designated as *AarJAZ1* to *AarJAZ18*, based on their chromosomal distribution (Table 1). The lengths of the amino acid sequences ranged from 162 to 318 amino acids (aa), with an average length of 221 aa. The molecular weights varied between 17.56 and 33.54 KDa, with an average of 28.23 kDa, and the isoelectric points (pI) ranged from 5.16 to 9.52. Among the 18 *A. argyi* JAZ proteins, only AarJAZ2, AarJAZ11, and AarJAZ15 were classified as acidic proteins (pI < 7), while the remaining 15 were basic proteins (pI > 7). The instability coefficients varied from 37.8 to 67.19, with AarJAZ8 being the only protein with an instability coefficient below 40. The other 17 AarJAZ proteins exhibited instability coefficients greater than 40, indicating potential instability [37]. Regarding hydrophobicity, the grand average of hydropathy (GRAVY) values for all 18 AarJAZ protein sequences were negative, suggesting that these proteins are hydrophilic. Predictions of subcellular localization indicated that all 18 AarJAZ proteins are localized in the nucleus, implying that they may function as transcription factors.

### 3.2. Phylogenetic Analyses and Classification of the A.argyi JAZ Gene Family

**Figure 1:**
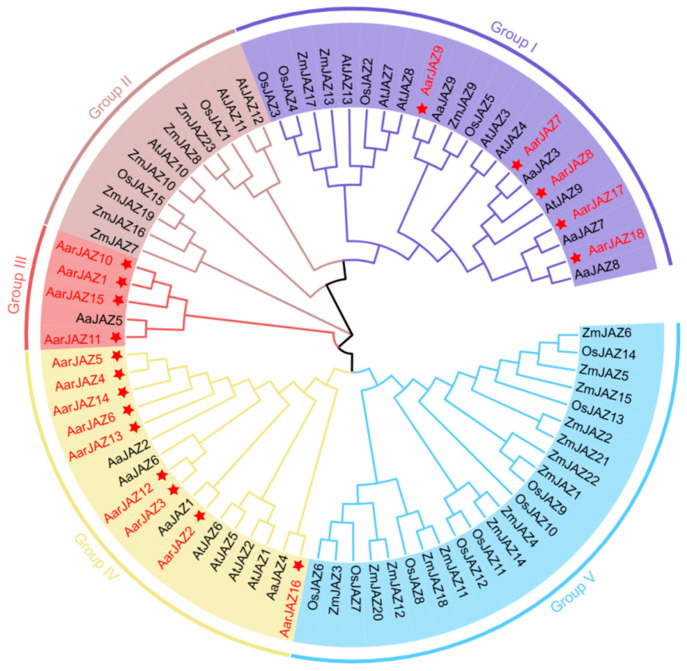
Phylogenetic tree of JAZ proteins from *A. thaliana, A. argyi, A.annua, O. sativa,* and *Z. mays.* The tree was constructed using MEGA11 by the neighbor-joining method, with 1000 bootstrap replicates.

To investigate the systematic evolutionary relationships of the *JAZ* gene family in *A. argyi*, we aligned 60 JAZ protein sequences using MEGA11 software, including 13 from *A. thaliana*, 9 from *A. annua*, 15 from *O. sativa*, and 23 from *Z. mays*. A phylogenetic tree was then constructed using the neighbor-joining method, which does not rely on complex evolutionary models and can infer phylogenetic relationships based on sequence distance. For our gene family data, the neighbor-joining method effectively reflects gene similarities and differences, and the resulting tree is easy to interpret. Based on this phylogenetic tree (Figure 1), the JAZ protein sequences were classified into five subfamilies. Notably, members of the *A. argyi JAZ* gene family are distributed across Groups I, III, and IV, with no members found in Groups II and V, which are predominantly populated by *JAZ* gene family members from monocot plants such as *Z. mays* and *O. sativa*. Group IV contains the largest number of members, with nine *A. argyi JAZ* genes, while Groups I and III contain five and four members, respectively. Additionally, the distribution pattern indicates that the *A. argyi JAZ* family members are closely related to those of *A. annua*, suggesting a high degree of homology between the *JAZ* gene families of these two species.

### 3.3. Conserved Motifs, Conserved Structural Domains, and Gene Structure Analysis of the JAZ Gene Family in A. argyi

**Figure 2:**
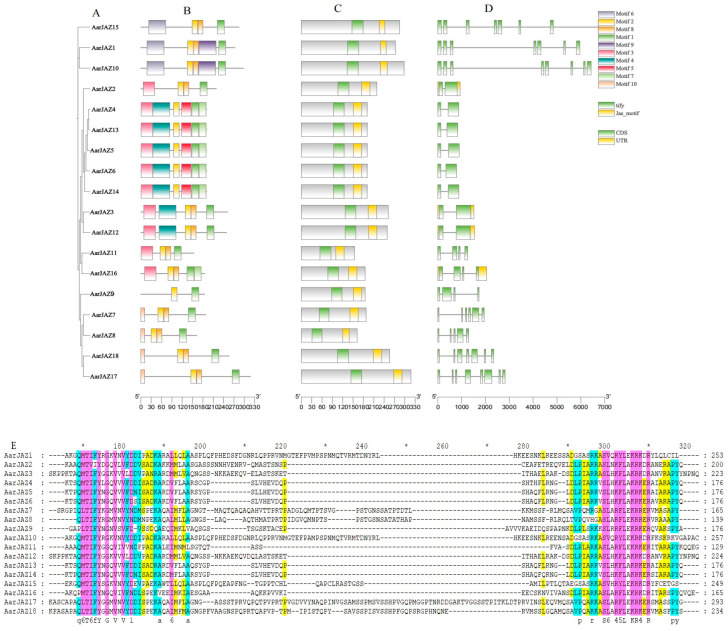
Phylogenetic relationship, conserved motif, and gene structure analyses of AarJAZs. (**A**) Phylogenetic tree. (**B**) Distribution of conserved domains in AarJAZs. Boxes of ten different colors represent different conserved domains. (**C**) Prediction and analysis of conserved domains in AarJAZs through NCBI-CDD. (**D**) Exon/intron distribution of AarJAZs. (**E**) Sequence alignment of TIFY and Jas domains in AarJAZs.

To further investigate the structural features and conserved domains of JAZ proteins, we analyzed 18 JAZ protein sequences using the MEME Suite, identifying 10 conserved motifs and predicting additional motifs in the JAZ proteins, which were then visualized (Figure 2B). Detailed motif information is provided in Table S2. A search of the NCBI-CDD database revealed that all JAZ protein sequences from *A. argyi* contain the conserved TIFY and CCT_2 domains. Further analysis of the conservation patterns of the TIFY and Jas domains across the 18 JAZ proteins was conducted using GeneDoc2.7 software, and the results indicated that these domains are not entirely conserved (Figure 2E).

Intron-exon structure analysis was performed to assess the diversity of the *A. argyi JAZ* gene architecture. As shown in Figure 2A, the number of exons in these *JAZ* genes varies from two to eight, with the majority (six genes) containing two introns, including *AarJAZ2*, *AarJAZ4*, *AarJAZ5*, *AarJAZ6*, *AarJAZ13*, and *AarJAZ14*. Notably, only two *JAZ* genes, *AarJAZ10* and *AarJAZ17*, contain eight exons. Additionally, it was observed that only *AarJAZ2*, *AarJAZ3*, *AarJAZ12*, and *AarJAZ16* possess untranslated regions (UTRs) at both the 5′ and 3′ ends (Figure 2D). Among the 18 AarJAZ proteins, 10 conserved motifs were identified, with each protein containing 2 to 6 motifs. Most members of the same subfamily exhibited a similar number and type of motifs, with similar distribution patterns, suggesting that proteins within the same subfamily may share functional similarities (Figure 2B). Motif1 and Motif2 were present in all 18 protein sequences, indicating that these motifs are widely distributed and highly conserved across the AarJAZ proteins. Additionally, members of the same subfamily showed similar distributions of the TIFY and Jas domains (Figure 2C). Finally, sequence alignments conducted using GeneDoc software revealed that the TIFY and Jas domains of the 18 JAZ proteins from *A. argyi* are poorly conserved (Figure 2E). For example, the TIFY domain in AarJAZ2 is represented as TVIY, and the Jas domain in AarJAZ1, AarJAZ5, and AarJAZ10 exhibited partial amino acid deletions (notably lacking the PY motif).

### 3.4. Chromosome Distribution of the JAZ Gene Family in A. argyi

**Figure 3:**
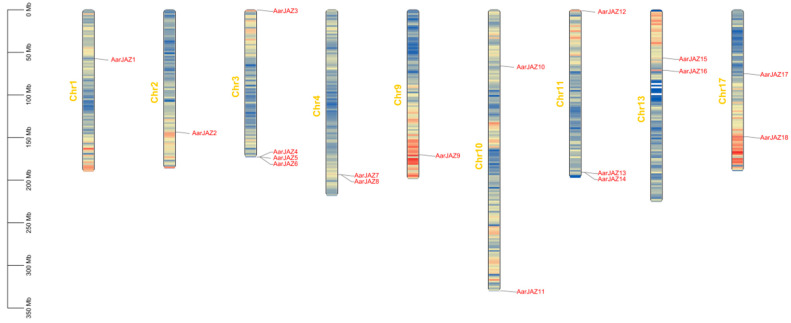
Chromosomal localization distribution of *AarJAZs*.

Chromosomal localization analysis of members of the *JAZ* gene family indicates that 18 *AarJAZ* genes are distributed among 9 of the 17 chromosomes. The distribution of *AarJAZ* genes across chromosomes is not uniform, with Chr3 containing the most *JAZ* genes (four genes), Chr11 containing three *JAZ* genes, Chr4, Chr10, Chr13, and Chr17 each containing two *JAZ* genes, and the remaining Chr1, Chr2, and Chr9 each containing one *JAZ* gene. This distribution pattern suggests that there is no significant correlation between chromosome length and the distribution of *AarJAZ* genes (Figure 3).

### 3.5. Analysis of Cis-Acting Elements in AarJAZ Promoters

**Figure 4:**
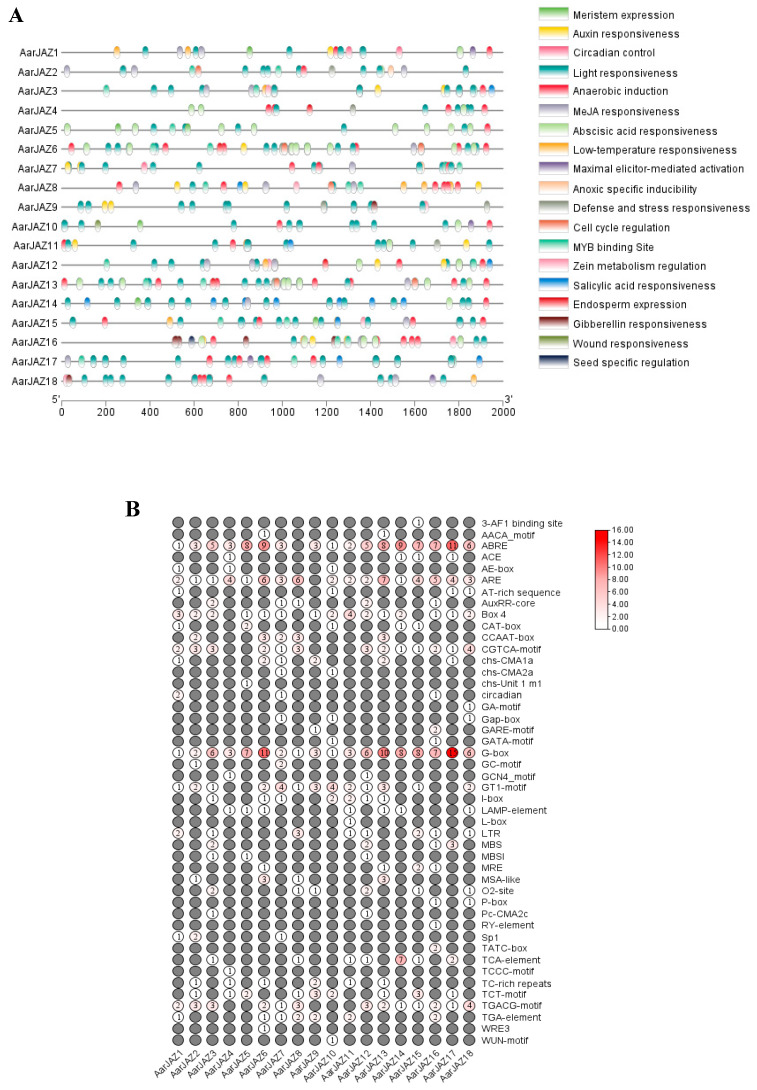
Predicted cis-elements in *AarJAZ* gene promoters. (**A**) The distribution and functional classification of the cis-acting elements 2000 bp upstream from the *JAZs*. (**B**) The numbers of each cis-acting element in the *JAZ* gene promoter.

Cis-acting elements serve as molecular switches that are closely associated with the regulation of gene expression under both biotic and abiotic stresses [38]. The analysis of cis-elements warrants further functional investigation of the *A. argyi JAZ* gene family. To this end, the 2000-bp upstream region of the *JAZ* genes was extracted for cis-element identification. The results revealed that the largest numbers of photoresponsive elements were located within the promoter regions of the *JAZ* genes, with the G-box element being widely present in various *JAZ* genes (Figure 4A). This suggests that *JAZ* genes may be regulated by light. Furthermore, many *JAZ* genes were found to harbor numerous cis-acting elements associated with hormone responses, including ABA response elements (ABRE), JA response elements (the TGACG and CGTCA motifs), IAA response elements (the TGA-element), GA response elements (P-box, GARE-motif, and TATC-box), and SA response elements (the TCA-element). Additionally, the *JAZ* genes exhibited a significant number of cis-elements that are involved in stress responses (Figure 4B), such as low-temperature-responsive elements (LTR), drought-responsive elements (MBS), and defense/stress-responsive elements (TC-rich repeats), among others. These findings suggest that the *A. argyi JAZ* genes play a crucial role in plant growth and development, in regulating hormonal signaling networks, and in mediating responses to both biotic and abiotic stresses.

### 3.6. Intraspecific Collinearity Analyses of the JAZ Gene Family in A. argyi

**Figure 5:**
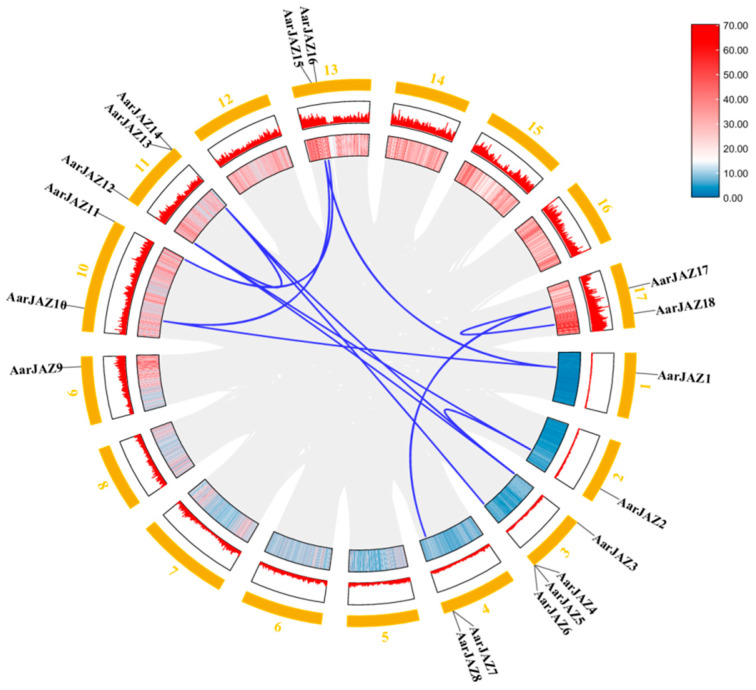
Synteny analysis of *AarJAZs*. The colors represent gene density, and the outermost numbers represent the individual chromosomes. Red means high gene density, blue means low gene density. The blue lines indicate segmentally duplicated gene pairs.

Gene duplication events contribute to the expansion of gene families and play a critical role in evolutionary adaptation by facilitating the acquisition of new gene functions. Given the significance of gene duplication in the evolution of plant gene families, we investigated the duplication patterns of the 18 *JAZ* family genes in the *A. argyi* genome and identified 12 pairs of homologous duplicates (Figure 5). To assess the evolutionary rate and selective pressures acting on the *JAZ* gene family in *A. argyi*, we performed Ka and Ks analyses (Table 2). The results revealed that the Ka/Ks ratios for all twelve pairs of duplicates were less than 1, indicating that these 12 pairs of *JAZ* genes in *A. argyi* have undergone a strong purifying selection process.

### 3.7. Expression Analysis and Gene Ontology Enrichment of the JAZ Gene Family in A. argyi

**Figure 6:**
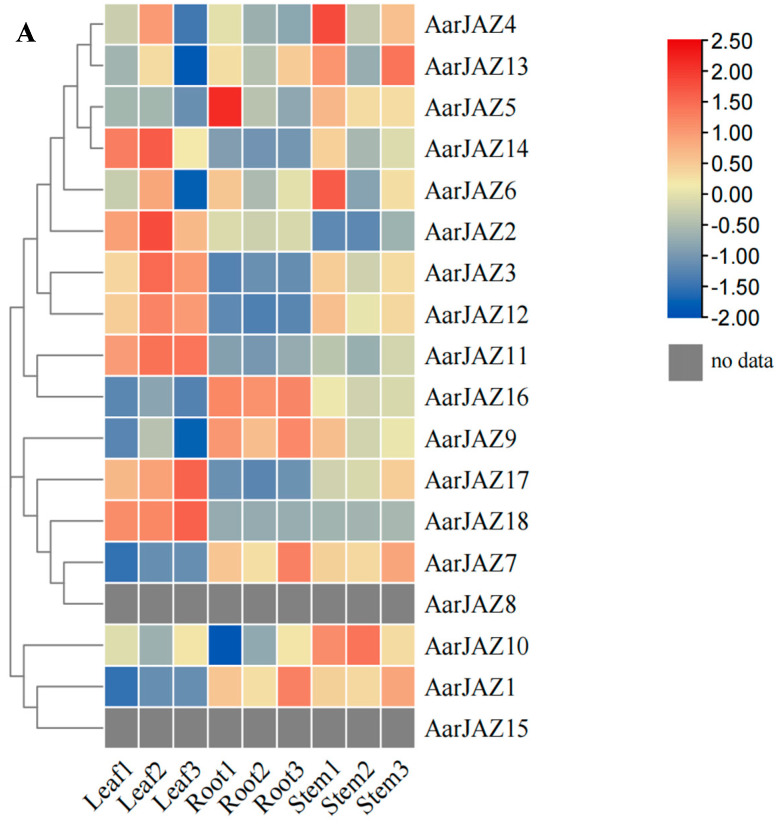

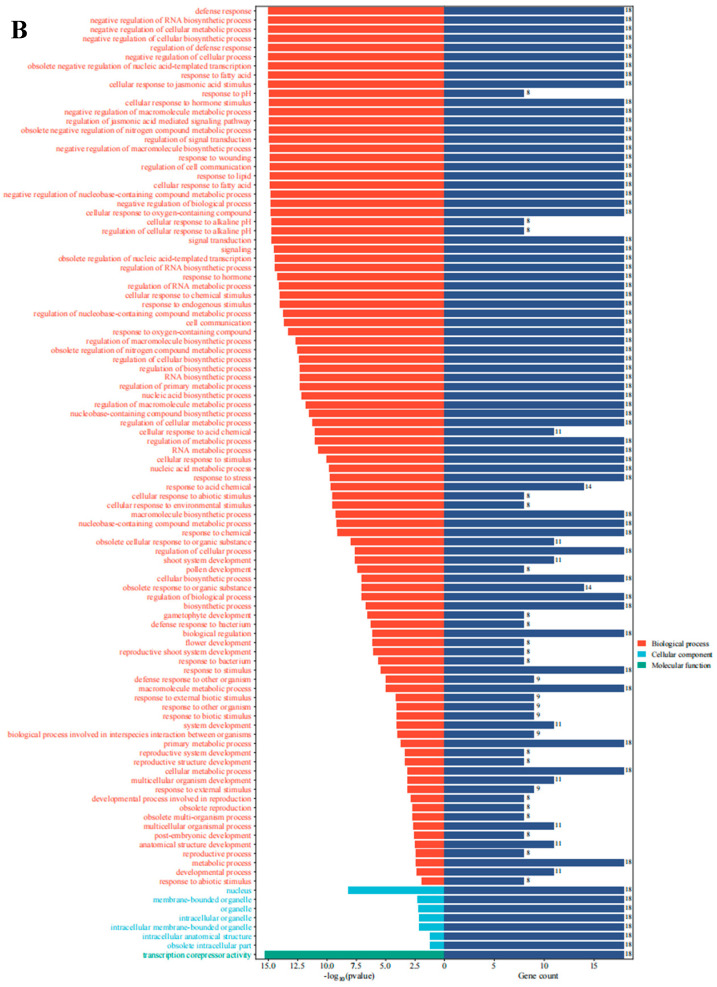
Expression analysis and gene ontology enrichment of *AarJAZs*. (**A**) Expression analysis of *AarJAZs* in different tissues. The expression level was calculated according to the FRKM. Red rectangles indicate high expression, while the blue rectangles represent low expression. (**B**) Gene ontology enrichment of *AarJAZs*.

To investigate the expression patterns of *AarJAZ* genes in different tissues, we performed an expression analysis of the *JAZ* gene family in *A. argyi* using publicly available transcriptome data from various tissues (root, stem, and leaf) [36]. Among the transcriptome data, we identified 18 homologous *JAZ* gene sequences. After performing BLAST filtering, 15 genes with a sequence alignment identity greater than 95% were selected. The expression levels of these genes were visualized using a clustering heatmap, with different colors representing varying expression intensities. The results (Figure 6A) showed that the expression of *AarJAZ14*, *AarJAZ2*, *AarJAZ3*, *AarJAZ12*, *AarJAZ11*, *AarJAZ17*, and *AarJAZ18* in the leaf tissues was significantly higher than in the stem and root tissues.

To further investigate the functional roles of *AarJAZ*s in biological processes, we conducted a GO enrichment analysis of 18 *JAZ* genes. The results revealed that these 18 genes were significantly enriched in 104 GO terms (Table S3). Of these, the majority (96 terms) were categorized as biological processes, while 7 terms were associated with cellular components and 1 with molecular function (Figure 6B). In the biological process category, key terms included stress response, hormone signaling, and negative regulation. Regarding cellular components, the prominent categories included the nucleus, membrane-bounded organelles, organelles, and intracellular organelles. In terms of molecular function, the only enriched term was transcription corepressor activity.

### 3.8. Screening of Candidate AarJAZ Genes for Glandular Trichome Development and the Relative Expression Levels in Different Tissues

In recent years, *JAZ* genes have been found to regulate the development of glandular trichomes in plants. In *A. thaliana*, JAZ proteins regulate the jasmonic acid (JA)-mediated initiation of non-glandular trichomes by inhibiting the formation of the WD-repeat/bHLH/MYB complex [39]. In *Nicotiana tabacum*, the overexpression of *NbJAZ3* has been shown to increase trichome density [40]. In *A. annua*, *AaJAZ8* has been demonstrated to inhibit the transcriptional activity of *AaHD1* and *AaGL3* through protein interactions, thereby influencing glandular trichome density [16,41]. In *Gossypium hirsutum*, *GmJAZ2* negatively regulates fiber initiation by interacting with the R2R3-MYB transcription factor *GhMYB25-like* [42]. These findings highlight the critical role of JAZ proteins in the molecular mechanisms underlying glandular trichome development.

**Figure 7:**
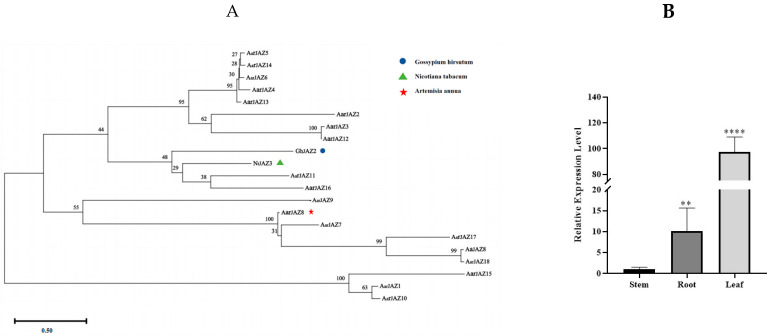
Phylogenetic tree of AaJAZS in *A. argyi* and JAZs in other species, and the qRT-PCR analysis of *AarJAZ18* in different tissues. (**A**) Phylogenetic tree of the AaJAZS, NbJAZ3, AaJAZ8, and GmJAZ2 proteins. (**B**) qRT-PCR analysis of AarJAZ18 in different tissues. Statistically significant differences were assessed using Student’s *t*-test (** *p* < 0.01, **** *p* < 0.0001).

To further investigate the functional similarity between JAZ proteins, we compared the reported protein sequences of *AaJAZ8*, *GhJAZ2*, and *NtJAZ3* with the *A. argyi* JAZ protein sequences using MEGA11 (Figure 7A). Our results reveal that *AarJAZ18* shares a close evolutionary relationship with *AaJAZ8*. Sequence alignment via NCBI BLAST showed that the amino acid sequence identity between AarJAZ18 and AaJAZ8 is as high as 98%. This suggests that *AarJAZ18* is likely to have a similar function to *AaJAZ8*; therefore, *AarJAZ18* may play a comparable role in regulating glandular trichome development. We further validated the expression levels of *AarJAZ18* in various organs using qRT-PCR. The results (Figure 7B) indicated that *AarJAZ18* was predominantly expressed in the leaves, which is consistent with the transcriptome data, further supporting the potential role of this gene in the glandular trichomes of *A. argyi* leaves.

## 4. Discussion

JAZ proteins are plant-specific and play a crucial role in various physiological processes, including plant growth and stress responses, through their involvement in the JA signaling pathway. However, research on JAZ proteins has primarily focused on model plants and studies on JAZ proteins within the genus *Artemisia* are limited, with reports mainly confined to *A. annua* [15]. In *A. argyi*, the lack of comprehensive genomic and transcriptomic data has resulted in only a few gene families being characterized. It was not until 2022 and 2023 that two genomic datasets for *A. argyi* were published [31,43]. Therefore, this study utilized bioinformatics tools to identify 18 *AarJAZ* genes in the *A. argyi* genome; it was also found that the conserved motifs in the protein sequences of these genes are not completely conserved. These amino acid variations may contribute to functional differences, suggesting that distinct JAZ proteins in *A. argyi* may have divergent roles. The analysis of these genes and proteins offers a theoretical foundation for future research on the functions of *JAZ* genes in *A. argyi*.

Gene duplication events have significantly contributed to the formation of new gene family members throughout the evolutionary history of plants. These events facilitate the generation of new genes during plant genome evolution, thereby enhancing the adaptability of plants to their environments. Gene duplication is also considered a key driver of plant evolution and innovation, providing an essential genetic source for the evolution of novel functions [44]. Consequently, the study of gene duplication events deepens our understanding of the processes underlying gene and species evolution. Whole-genome duplication, segmental duplication, and tandem duplication are the primary mechanisms of gene duplication [45]. Segmental duplication plays a dominant role in the generation and maintenance of gene families, serving as the main source of gene structural variation and innovation [46]. This study identified 18 *AarJAZ* genes within the *A. argyi* genome. Through collinearity analysis, we determined that 13 of these genes originated from segmental duplication, while the remaining 5 were generated by tandem or whole-genome duplication. These findings suggest that the abundant *JAZ* genes in *A. argyi* are primarily the result of segmental duplication, a mechanism also observed in species such as *Petunia hybrida* (morning glory) and *Raphanus sativus* (radish) [47,48]. Furthermore, the diversity of conserved protein domains among the 18 AarJAZ proteins not only indicates a high degree of evolutionary refinement in the JA signaling pathway of *A. argyi* but also suggests the significant adaptability of *A. argyi* to its environmental conditions.

Transcription factors regulate gene expression by interacting with the promoter regions of target genes, which are crucial for these regulatory processes [49]. Thus, analyzing the cis-acting elements in the promoter regions of the *JAZ* genes in *A. argyi* provides valuable insights into their potential functions. Our analysis of the cis-acting elements in the *A. argyi JAZ* genes revealed a high abundance of light-responsive elements, suggesting that *JAZ* genes may play a significant role in light-regulated plant development. Additionally, we identified numerous hormone-responsive elements, including those for abscisic acid (ABA), indole-3-acetic acid (IAA), salicylic acid (SA), gibberellic acid (GA), and jasmonic acid (JA). These findings highlight the involvement of *A. argyi JAZ* genes in hormone-mediated growth and development. The presence of multiple stress-responsive elements further supports their essential role in stress responses. GO enrichment analysis further emphasizes that *JAZ* genes are closely associated with stress responses, hormone signaling, and negative regulation. Moreover, these genes are predominantly localized in the nucleus, where they exert negative regulatory effects on the expression of target genes through interactions with various transcription factors.

Among the species in the genus Artemisia, *A. argyi* is one of the most well-known. The 2015 Nobel Prize in Physiology or Medicine was awarded for the discovery of artemisinin, an antimalarial compound isolated from *A. annua*, which has spurred global interest in the study of other *Artemisia* species [50,51]. In *A. annua*, much of the research has focused on increasing artemisinin production by enhancing the density of glandular trichomes [52,53,54]. This research provides valuable insights for trait improvement and cultivar breeding in *A. argyi*, where glandular trichomes are key sites for the synthesis and storage of essential oils, as well as metabolites such as flavonoids and terpenoids. Thus, increasing glandular trichome density in *A. argyi* could enhance the production of secondary metabolites, thereby increasing the medicinal value of the plant. In this study, we constructed a phylogenetic tree of several JAZ proteins that are known to regulate trichome development (Figure 6). We identified *AarJAZ18*, which is highly homologous to *AaJAZ8*, with an amino acid sequence similarity of 98%. This suggests that *AarJAZ18* may function similarly to *AaJAZ8* in regulating protein function. Based on these findings, we propose that *AarJAZ18* is a key candidate gene involved in the regulation of glandular trichome development in *A. argyi* leaves. Further tissue-specific expression analysis and subcellular localization studies revealed that *AarJAZ18* is primarily localized in the nucleus and is most highly expressed in the leaves, suggesting that *AarJAZ18* may function as a transcription factor regulating trichome development in medicinally important leaf tissues. Although no effective transgenic transformation system for *A. argyi* has been reported so far, we believe that developing such a system is crucial for further investigating the role of *AarJAZ18* in *A. argyi*. Future research will focus on constructing a reliable transformation system to validate our hypothesis. Additionally, potential experiments could include the investigation of *AarJAZ18*’s interactions with other transcription factors and its response to environmental stressors. Further exploration into its regulatory mechanisms under different developmental stages and stress conditions would provide more comprehensive insights. Expanding on tissue-specific expression patterns and functional validation through transgenic models would be key to elucidating the precise role of *AarJAZ18* in glandular trichome development. These studies could significantly contribute to advancing breeding strategies aimed at enhancing the medicinal properties of *A. argyi*.

## 5. Conclusions

*JAZ* genes are known to play crucial roles in various aspects of plant growth and development. However, the identification and functional analysis of JAZ proteins in *A. argyi* have not yet been explored. In this study, we have identified 18 putative *JAZ* genes in *A. argyi*, expanding our understanding of the JAZ gene family in this species. Phylogenetic analysis revealed that *AarJAZs* are divided into three subfamilies (subfamilies I, III, and IV), shedding light on their evolutionary relationships. Our promoter cis-element analysis demonstrated that *AarJAZs* are regulated by light, multiple plant hormones, and stress factors, indicating that these genes may play an important role in *A. argyi*’s stress tolerance mechanisms. Further functional insights, derived from GO enrichment analysis, suggest that *AarJAZs* are integral to stress responses, hormone signaling, and negative regulation, and are primarily localized in the nucleus. These findings imply that *AarJAZs* may act as negative regulators by interacting with the transcription factors involved in these processes. Notably, our study highlights *AarJAZ18* as a potential key regulator in glandular trichome development, which could have significant implications for the breeding and genetic improvement of *A. argyi*. Moving forward, further functional characterization of these genes, particularly in relation to glandular trichome development and stress responses, would be crucial to validate their roles and explore their potential for crop improvement. Additionally, investigating the interactions between *AarJAZs* and other transcription factors under different environmental conditions will provide further insights into their regulatory networks.

## Figures and Tables

**Table 1:** Physiochemical parameters and subcellular location of the 18 *JAZ* genes in *A. argyi*.

**Gene Name**	**AA (aa)**	**Mw (KDa)**	**pI**	**Instability Index**	**Aliphatic Index**	**GRAVY**	**Subcellular Localization**
*AarJAZ1*	273	30.77	7.71	64.1	75.71	−0.626	Nucleus
*AarJAZ2*	219	24.24	6.32	42.03	68.22	−0.521	Nucleus
*AarJAZ3*	252	27.78	8.82	41.37	75.91	−0.658	Nucleus
*AarJAZ4*	191	21.34	9.01	47.3	83.19	−0.414	Nucleus
*AarJAZ5*	191	21.35	9.46	44.76	75.08	−0.49	Nucleus
*AarJAZ6*	191	21.30	9.54	45.68	78.12	−0.483	Nucleus
*AarJAZ7*	188	20.41	9.15	45.85	54.04	−0.669	Nucleus
*AarJAZ8*	162	17.56	9.00	37.8	54.94	−0.809	Nucleus
*AarJAZ9*	185	20.14	9.52	67.19	78.05	−0.471	Nucleus
*AarJAZ10*	298	33.05	8.64	67.06	61.24	−0.886	Nucleus
*AarJAZ11*	154	16.92	5.16	41.11	65.39	−0.457	Nucleus
*AarJAZ12*	249	27.37	8.69	40.69	75.66	−0.637	Nucleus
*AarJAZ13*	191	21.19	9.23	40.72	78.64	−0.432	Nucleus
*AarJAZ14*	191	21.40	9.34	50.56	72.51	−0.542	Nucleus
*AarJAZ15*	285	31.32	5.98	48.99	69.47	−0.529	Nucleus
*AarJAZ16*	185	20.45	9.39	59.77	73.24	−0.464	Nucleus
*AarJAZ17*	318	33.54	9.10	46.49	63.81	−0.361	Nucleus
*AarJAZ18*	273	27.79	7.71	64.1	75.71	−0.626	Nucleus

aa: amino acid, KDa: kilodaltons.

**Table 2:** Estimated Ka/Ks ratios of the duplicated *JAZ* genes in *A. argyi*.

Gene Name		Ka	Ks	Ka/Ks
*AarJAZ1*	*AarJAZ10*	0.041837564	0.093916942	0.445474085
*AarJAZ1*	*AarJAZ15*	0.280459956	0.665372721	0.421508047
*AarJAZ2*	*AarJAZ3*	0.426632786	1.499285737	0.284557357
*AarJAZ2*	*AarJAZ12*	0.415204839	1.518940417	0.27335163
*AarJAZ3*	*AarJAZ12*	0.008765622	0.0611648	0.143311547
*AarJAZ3*	*AarJAZ13*	0.361421188	2.108519358	0.171409945
*AarJAZ4*	*AarJAZ13*	0.049979758	0.104334602	0.479033391
*AarJAZ7*	*AarJAZ17*	0.337025619	1.109457188	0.303775236
*AarJAZ10*	*AarJAZ15*	0.300098529	0.674987784	0.44459846
*AarJAZ11*	*AarJAZ16*	0.386487979	2.248902993	0.171856225
*AarJAZ12*	*AarJAZ13*	0.347084078	1.978497747	0.175428088
*AarJAZ17*	*AarJAZ18*	0.242598965	1.165132527	0.208215769

## Data Availability

Data are contained within the article.

## Supplementary Materials

The following supporting information can be downloaded at: https://www.mdpi.com/article/10.3390/cimb47020100/s1, Table S1: Protein sequences of *JAZ* genes in *A. argyi*; Table S2: Motif logos and sequences of Motifs 1–10, obtained using the MEME online website; Table S3: Gene ontology enrichment of the JAZ gene family; Table S4: Protein sequences of the *JAZ* genes in *Arabidopsis*, *Artemisia annua*, *Oryza sativa*, and *Zea mays*; Table S5: List of primers used in this study.
